# ZnO Nanoparticles Obtained by Green Synthesis as an Alternative to Improve the Germination Characteristics of *L. esculentum*

**DOI:** 10.3390/molecules27072343

**Published:** 2022-04-06

**Authors:** David Asmat-Campos, Eloy López-Medina, Gabriela Montes de Oca-Vásquez, Efraín Gil-Rivero, Daniel Delfín-Narciso, Luisa Juárez-Cortijo, Luigi Villena-Zapata, Julio Gurreonero-Fernández, Roly Rafael-Amaya

**Affiliations:** 1 Dirección de Investigación, Innovación & Responsabilidad Social, Universidad Privada del Norte (UPN), Trujillo 13011, Peru; 2 Grupo de Investigación en Ciencias Aplicadas y Nuevas Tecnologías, Universidad Privada del Norte (UPN), Trujillo 13011, Peru; daniel.delfin@upn.edu.pe (D.D.-N.); luisa.juarez@upn.edu.pe (L.J.-C.); 3 Laboratorio de Biotecnología del Instituto de la Papa y Cultivos Andinos, Facultad de Ciencias Biológicas, Universidad Nacional de Trujillo, Av. Juan Pablo II s/n., Ciudad Universitaria, Trujillo 13011, Peru; slopezm@unitru.edu.pe (E.L.-M.); arivero@unitru.edu.pe (E.G.-R.); jory2599@gmail.com (R.R.-A.); 4 National Laboratory of Nanotechnology, National Center for High Technology, Pavas, San José 10109, Costa Rica; gmontesdeoca@cenat.ac.cr; 5 Campus Virtual, Universidad Privada del Norte (UPN), Trujillo 13011, Peru; luigi.villena@upn.pe; 6 Facultad de Ingeniería, Universidad Privada del Norte (UPN), Av. Del Ejército 920, Trujillo 13006, Peru; julio.gurreonero@upn.edu.pe

**Keywords:** nanoparticles, nanofertilizer, zinc, green synthesis, germinative characteristics, tomato

## Abstract

Tomato is an important crop due to its nutritional contributions and organoleptic properties, which make it an appetizing vegetable around the world. In its sowing, the use of seed is the most accessible propagation mechanism for farmers. However, the induction to germination and emergence is often limited in the absence of stimulants that promote the development and growth of the seedling, added to the interference of infectious agents that notoriously reduce the vitality and viability of the seed. Given this, it was proposed as a research objective to determine the effect of zinc oxide nanoparticles (ZnO NPs) mediated by a green route on the germinative characteristics of *Lycopersicon esculentum* Mill. 1768 “tomato”. The experimental phase consisted of the synthesis of ZnO NPs and its subsequent characterization. After its synthesis, its inoculation was conducted during the germination of seeds of *L. esculentum*, considering six sample groups for the treatment with zinc nanoparticles (T1: Control; T2: 21.31 ppm; T3: 33.58 ppm; T4: 49.15 ppm; T5: 63.59 and T6: 99.076 ppm). The results indicate that concentrations close to 100 ppm of ZnO NPs are ideal in the treatment of *L. esculentum* seeds, due to the promotion of enzymatic and metabolic activity to achieve cell elongation; likewise, the biosynthesized nanoparticles showed no phytotoxicity, due to the fact that, in all the treatments, there were processes of germination and emergence. This was linked to the generation of a Zn^0^-phenolate complex through a chelating effect, which generates compatibility with the seed and, compared to classic inorganic synthesis, usually shows phytotoxicity. In this sense, green synthesis is presented as a great alternative in this type of application.

## 1. Introduction

*L. esculentum*, belongs to the Solanaceae family. It is characterized by being an herbaceous species with hermaphroditic flowers and berry-type fruits [1]. It is currently considered a cosmopolitan crop with a high nutritional value because it provides numerous nutrients and phytochemical compounds with beneficial actions for health [2]. We highlight the presence of lycopene, which is a powerful hypocholesterolemic antioxidant. The β-carotene and lutein, which act as enhancers of the immune system with anticancer action, in addition to the presence of mineral salts and vitamins, adds to its pleasant organoleptic properties, makes it an appetizing functional food [3,4,5,6].

Due to the multiple applications of the *L. esculentum* fruit, its cultivation has become widespread around the world, reaching a world production of 34.32 million tons during 2018, with the United States as the main producer [7]. Therefore, aspects related to its propagation and cultivation are fundamental, especially those where hybrid seeds are used, which constitutes the alternative with the greatest access and ease for farmers. There are different mechanisms to induce germination, from the use of auxins to the use of green synthesis stimulants that seek to maximize the speed of germination, which prevents the intrusion of bacteria, viruses and fungi that markedly reduce the percentage of emergence [8,9,10].

Currently nanotechnology has been used in several applications and industries, including agriculture [11]. The synthesis of nanoparticles (NPs) has great potential in applications as agents that promote cell division and elongation and has an influence on the increased activity of antioxidant enzymes and reactivity of cytokinins and gibberellins, thereby, enhancing the germination process. In addition, it has been determined that they have bactericidal, fungicidal and virucidal actions on plant pathogens [12,13,14,15].

There is a great diversity of methods for the production of NPs, leading to a great diversity of sizes, shapes, and stability. Recent research is oriented to the production of NPs through green synthesis, in which environmentally friendly methods are used. Among the most widely used “green” methods are those that use extracts from the leaves, fruits, stems and barks of various plants, which replace the synthetic chemicals responsible for the reduction process of a precursor material, thus, being the most economical green methods and with less production of polluting waste [16,17,18,19].

Studies by Kumar et al. [20] have demonstrated the effectiveness of green synthesis method, by obtaining spherical silver nanoparticles (AgNPs) between 12 and 50 nm from the reduction of silver nitrate using blueberry extract. While Phongtongpasuk [21], using the pitahaya peel extract, synthesized silver nanoparticles between 25 and 26 nm. In turn, Luminita et al. [22] studied the reducing action of elderberry extract, obtaining AgNPs between 20 and 80 nm. Odeniyi et al. [23] synthesized 12 nm AgNPs from *Nauclea latifolia* extract, which were used for the formulation of antimicrobial products.

Advances in the applications of nanomaterials have made possible to demonstrate the action of NPs as mediators in the growth process of various plants [24,25,26]. Zinc oxide NPs (ZnO NPs) with an average size of 35 nm obtained by green synthesis, were shown to improve root and shoot growth in *Triticum aestivum L*. plants under different concentrations. The concentration of 62 mg/L of ZnO NPs was the one that provided a better growth [27].

Likewise, the action of the ZnO NPs at concentrations of 1, 3, 5, 7 and 9 mg/mL on the growth of *Sesamum indicum* was evaluated, determining that under a concentration of 5 mg/mL growth was stimulated, while for higher concentrations, a reduction in both root and shoot size is observed; this allows indicating that ZnO NPs can be a potential alternative source to be used as nanofertilizer [28]. Mendez et al. [13] used ZnO NPs, and a higher growth and dry biomass production in *C. annuum* plants was determined due to a higher enzymatic activity. In turn, Estrada [29], corroborated, using infrared spectroscopy, that the use of ZnO NPs generates a greater production of carbohydrates, proteins and lipids in seedlings of *Zea mays* L.

Given the need for greater knowledge about the efficiency of nanoparticles in a higher rate of germination and viability in highly demanded agricultural products and, at the same time, sensitive to a series of microorganisms, it was proposed as a research objective to determine the effect of nanoparticles of zinc produced by a green synthesis method on the germinative characteristics of *L. esculentum*.

## 2. Materials and Methods

### 2.1. Green Synthesis and Characterization of Zinc Oxide Nanoparticles (ZnO NP)

For this procedure, a green synthesis method was applied using *Coriandrum sativum* extract as the organic reducer. For the elaboration of the extract in aqueous solvent, fresh leaves were collected from a local supermarket in La Libertad region, Peru, which were washed three times with ultrapure water to eliminate any type of impurities, subsequently dried at 40 °C for 10 h, crushed and sieved. Then, the mixture of 30 g of dry powder in 500 mL ultrapure water was made by magnetic stirring (T° = 70 °C at 400 RPM) for 4 h. Filtering was done with a diaphragm vacuum pump (GAST DOA-P704-AA). The extract was kept refrigerated (5 °C) for future synthesis.

For the synthesis of ZnO NPs, the precursor zinc acetate dihydrate was used (CH_3_COO)_2_Zn2H_2_O) (Merck Millipore, Burlington, MA, USA, CAS no. 5970-45-6) at a concentration of 0.21 M, which was diluted in ultrapure water and placed under magnetic stirring (600 RPM) until reaching a temperature of 70 °C, and then 20 mL of previously prepared aqueous extract of *C. sativum* was added dropwise, maintaining the same stirring and temperature parameters for 90 min. After that, the mixture was placed in a muffle oven for 5 h at 500 °C for calcination. Finally, the sample was ground using a mortar, until obtaining a white powder, characteristic of this type of nanomaterial.

From the nanomaterial obtained (100 ppm), a total of five dilutions were made with ultrapure water to obtain the following concentrations: T2 = 21.35 ppm, T3 = 33.58 ppm, T4 = 49.15 ppm, T5 = 63.59 ppm and T6 = 99.076 ppm. Sample T1, corresponds to the control sample where only ultrapure water was used.

### 2.2. Characterization of ZnO NPs

The ZnO NPs were characterized by UV-vis Spectrophotometry (Hewlett Packard, 8452, Palo Alto, CA, USA) in a range of 380–800 nm, this as an initial analysis to determine the presence of the Surface Plasmon Resonance (SPR) peak. Characterization was performed by Fourier Transform Infrared Spectroscopy (FT-IR) (Nicolet iS50, Thermo Fisher Scientific) in the range of 480–4000 cm^−1^, to determine the presence of functional groups present in the nanomaterial, due to the process of green synthesis adopted in this investigation; additionally, the OMNIC 8.1 software was used for reading of the spectra.

The size and morphology were studied by Transmission Electron Microscopy (TEM) JEOL (JEM 2011 model) with a voltage acceleration of 12 KV; likewise, elemental analysis was performed by EDS in a Scanning Electron Microscopy (SEM) equipment (OXFORD EDS 6498). To know its crystalline structure, X-Ray Diffraction (XRD) analysis (Bruker, D8 Advance Eco) was performed in a range of 20° and 80° (2Theta) with a voltage of 30 KV and a current of 10 mA and 300 W power.

### 2.3. Germination of Seeds of L. esculentum

We randomly selected 720 seeds of *L. esculentum*, which were distributed in two batches, destined for germination and emergence. For sowing, Petri dishes were used, provided with eight filter paper disks, without treating the seeds with fungicides. For the six treatments and three repetitions each, 20 seeds were distributed per repetition, making a total of 360 seeds. A control T1 treatment was used that included only the addition of distilled water to the seeds. All treatments were kept under greenhouse conditions (20 ± 4 °C) for 7 days until seedling emergence.

For calculations of the germination indices, the following was determined: 1. The percentage of germination (%G): calculated based on the number divided by the total number of seeds sown, multiplied by 100. 2. The mean daily germination (MDG): the final percentage of germination divided by the number of days of curing of the test. 3. Czabator index (CI): Result of the multiplication of MDG × VA (highest value of the curve). 4. Emergence percentage (%E): obtained by dividing the number of seedlings emerged during the test with the total number of seeds sown, multiplied by 100 [30].

### 2.4. Inoculation of ZnO NPs in Seeds of L. esculentum

From six treatments, five were destined for treatment with ZnO NPs colloid (T2, T3, T4, T5 and T6). For this, 10 mL of nanoparticulate colloid (previously homogenized by ultrasound for 15 min) was added once (individually for each treatment) on the filter paper which contained the seeds of *L. esculentum*, the exposure to the ZnO NPs was for 24 h. Optimum temperature conditions (20 ± 4 °C) were also provided to favor germination.

### 2.5. Characterization by Atomic Absorption of Seedlings of L. esculentum

The elemental concentration (Zn) was determined with an Atomic Absorption Spectrophotometer (Agilent Technologies, 200 AA series), for which the samples were initially washed with distilled water, subsequently calcined (500 °C) for 2 h, and then carefully checked to avoid the presence of impurities. The ash samples were digested as follows: For the 12 coded samples (foliage + root), 0.5 g of ash was weighed, and its content was poured into each 100 mL beaker with 50 mL of ultrapure water for the dissolution of ash particles. Finally, 5 mL of nitric acid was added to each coded beaker for subsequent acid digestion in a hotplate. Once the digestion was completed and without the presence of impurities, the reading was conducted.

### 2.6. Statistical Analysis

For statistical analysis, the statistical software RStudio version 4.1.2 was used. The data of variables, germination percentage and emergence percentage were analyzed using the unifactorial ANOVA design due to compliance with homoscedasticity, comparing six treatments and 18 treatments. experimental units (petri dishes); thus, Tukey’s post hoc tests were also applied. For the variable zinc absorption in stem and root, Welch’s unifactorial ANOVA design was applied due to non-compliance with homoscedasticity, and Games Howell’s post-hoc test, for the selection of the best treatment.

## 3. Results

### 3.1. Characterization of ZnO NPs Mediated by Green Synthesis Using C. sativum Extract

**Figure 1:**
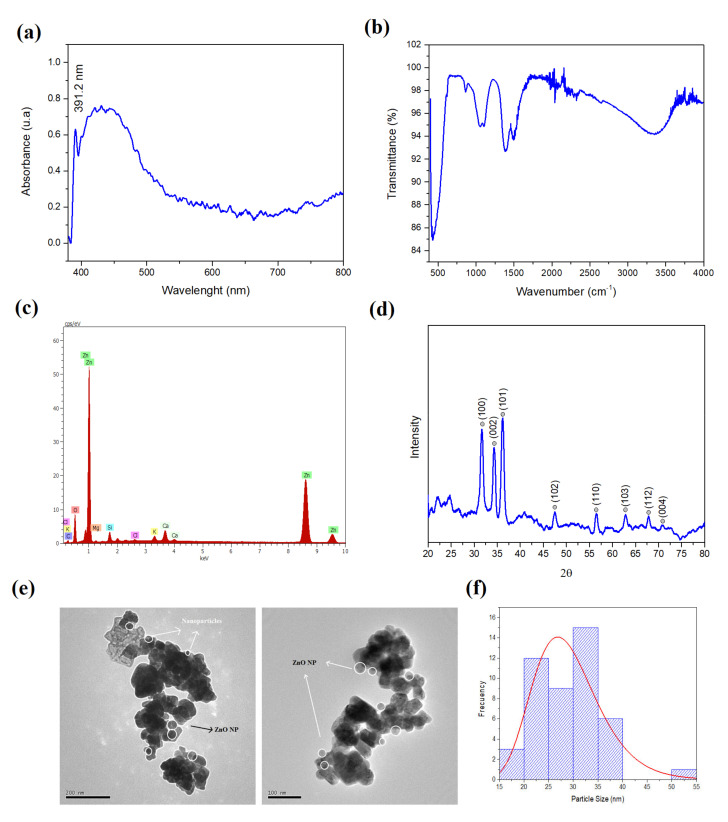
Characterizations of ZnO NPs, (**a**) UV-vis Spectrophotometry, (**b**) FT-IR Spectrum, (**c**) Elemental Analysis by EDS, (**d**) X-Ray Diffraction (XRD), (**e**) TEM Images and (**f**) histogram of nanoparticle sizes.

As mentioned in the methodology section, the standard sample of ZnO NPs (100 ppm) was characterized, a sample with which it was split to dilute with ultrapure water at different concentrations (ppm). In the characterization by UV-vis spectrophotometry (Figure 1a), the presence of the absorbance peak at 391.2 nm stands out, whose value is characteristic for this type of nanomaterial with a hexagonal-type structural configuration [31].

FT-IR characterization was also performed (Figure 1b), providing information on the presence of vibrations related to Zinc (Zn) and oxygen (O), where the peak at 429.5 cm^−1^ corresponds to the ZnO NPs [32,33,34]. Vibrations can be located at 1384 cm^−1^ corresponding to aromatic amides [35] and at 1494 cm^−1^ linked to amine NH vibrational stretching in amide bonds in protein. In addition, the elemental analysis determined by EDS (Figure 1c) shows well-defined peaks that corroborate the presence of Zn.

The evaluation of the crystal structure by XRD (Figure 1d) reinforces the presence of the ZnO NPs, since it shows the peaks at (100), (002), (101), (102), (110), (103), (112) and (004) attributed to the hexagonal phase of ZnO, this corroborated with the JCPDS file: 36-1451, where the respective patterns are included in the same diffractogram. The morphology and size of the NPs was evaluated by TEM (Figure 1e), where the presence of NPs with spherical geometry was determined, without the presence of densities that are linked to any organic trace from the extract. This is due to the nanoparticles being subjected to calcination. Figure 1f shows the histogram of sizes obtained from the TEM result, where polydispersity can be observed but with an average size of 30 nm.

### 3.2. Germination and Emergence

**Figure 2:**
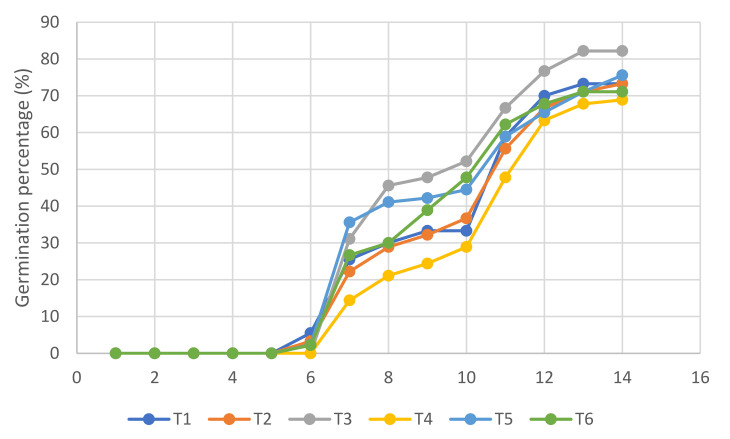
Germination curve of *L. esculentum*.

**Figure 3:**
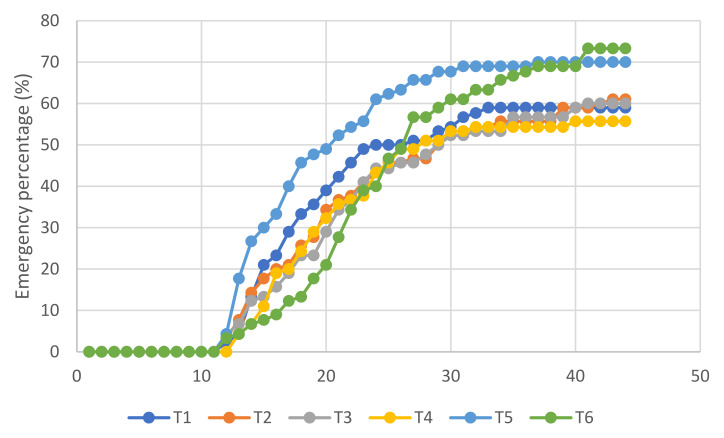
Emergence curve of *L. esculentum*.

The cumulative percentage curve of germination and emergence of *L. esculentum* (Figure 2 and Figure 3) was evaluated. Treatment 3 (T3) had a higher percentage of germination, which is reflected in a higher mean daily germination (MDG) and Czabator index (CI). However, treatment 6 (T6) obtained the highest percentage of emergence, which indicates the effect of the ZnO NPs.

**Figure 4:**
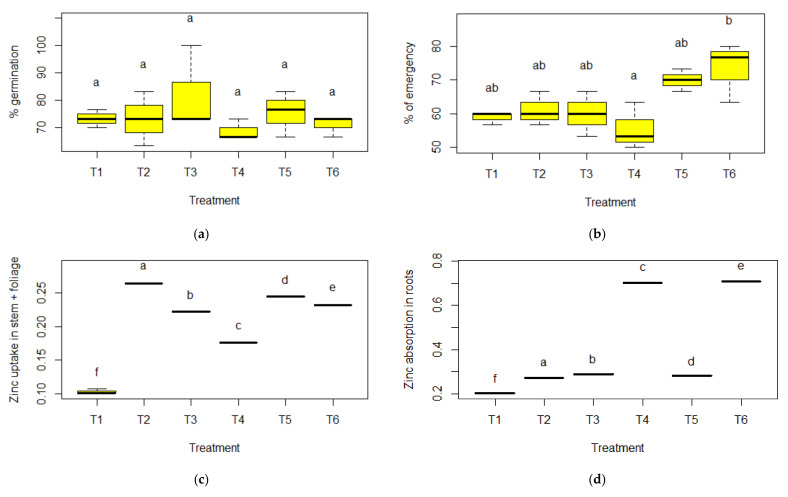

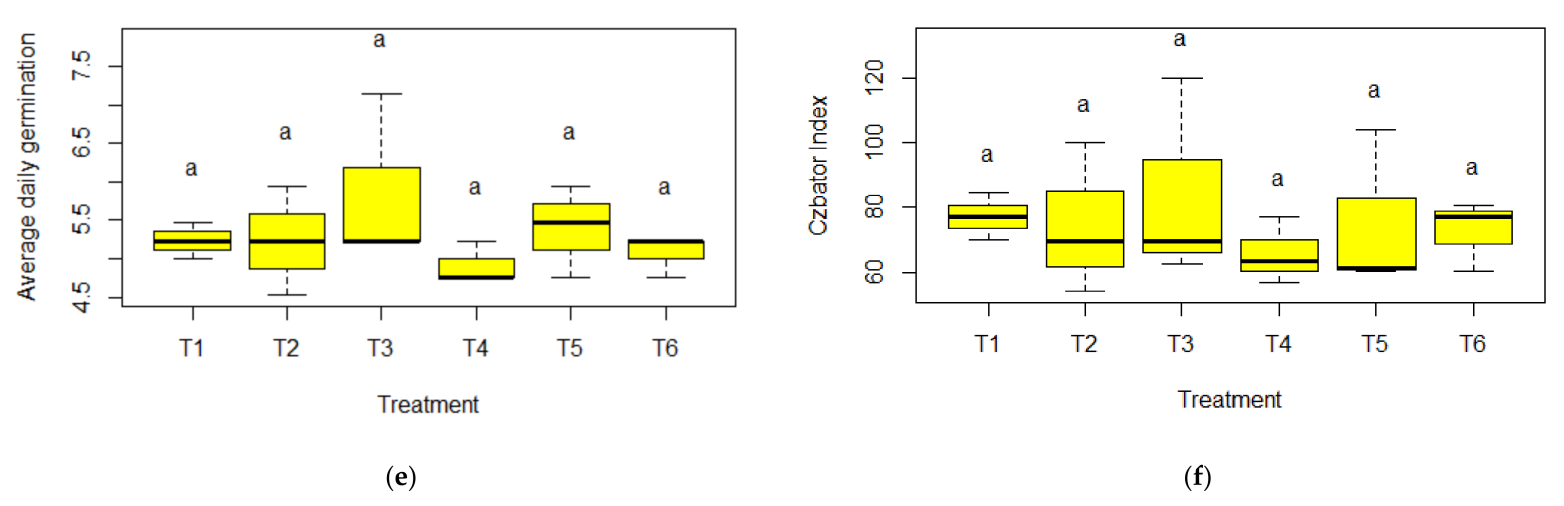
Statistical analysis of the study on the effect of zinc oxide nanoparticles mediated by green route on the germinative characteristics of *L. esculentum.* (**a**) Percentage of Germination, ANOVA, *p* = 0.540 and Post hoc Tukey’s test. (**b**) Percentage of Emergence, ANOVA, *p* = 0.021 and post hoc Tukey’s test. (**c**) Zinc absorption in Stem plus foliage, Welch’s ANOVA, *p* = 0.000 and Games–Howell post hoc test. (**d**) Root zinc absorption, Welch’s ANOVA, *p* = 0.000 and Post hoc Games–Howell test. (**e**) Mean daily germination, ANOVA, *p* = 0.544 and Post hoc Tukey’s test. (**f**) Czabator index, ANOVA, *p* = 0.843 and Post hoc Tukey’s test. Note: Means with different letters are significantly different, the horizontal lines across the box plots represent the medians, and the vertical lines protruding from the box correspond to the whiskers.

An in-depth statistical analysis (Figure 4) of the study linked to the effect of ZnO NPs on the germination characteristics of *L. esculentum* was performed with a 95.0% confidence level. According to the results, the ANOVA test of the complete random design for independent groups shown in Figure 4a, corresponding to the germination percentage variable, presented a *p*-value greater than 0.05 (*p* = 0.540 > 0.05); therefore, there is insufficient evidence to reject the hypothesis of equality of means $\left(H_{0}:\;\mu_{1}=\mu_{2}=\ldots =\mu_{6}\right)$, thus, concluding that there was no significant difference between treatments for the percentage of germination.

On the other hand, the *p*-value associated with the ANOVA test of the complete random design observed in Figure 4b, turned out to be less than 0.05 (*p* = 0.021 < 0.05) for the variable percentage of emergence, generating the rejection of the hypothesis of equality of means $\left(H_{0}:\;\mu_{1}=\mu_{2}=\ldots =\mu_{6}\right)$, suggesting that at least one of the means differs significantly from the others, for the variable percentage of emergence. In turn, Tukey’s post hoc test distinguishes different homogeneous groups; however, the treatment that presented the highest average in the emergency percentage variable corresponds to treatment T6 ($\bar{X}=73.333$); therefore, it is the best for the study of the effect of zinc nanoparticles of green synthesis on the germinative characteristics of *L. esculentum*. 

The significance of the *p*-value of the Welch’s ANOVA test presented a value less than 0.05 (*p* = 0.000 < 0.05), thus, rejecting the null hypothesis. Therefore, there was a significant effect of treatments on the variable absorption of zinc in stem plus foliage. The results of the Games–Howell post hoc test revealed that all treatments presented a significant difference (each treatment presented a different letter). Treatment 2 presented the highest average absorption of zinc followed by treatments 5 and 6.

The Welch’s ANOVA test, reached a *p*-value of significance less than 0.05 (*p* = 0.000 < 0.05); therefore, we concluded that there was a significant effect of treatments on the variable absorption of zinc in root; whereas, we can observe that the results obtained by the Games–Howell post hoc test, each treatment was found in a single homogeneous group, that is, there was a significant difference between the treatments, identifying that treatment 6 presented a higher average absorption of zinc in the roots.

The *p*-value of significance of the unifactorial ANOVA test shown in Figure 4e, presented a value greater than 0.05 (*p* = 0.544 > 0.05); therefore, there was no significant difference between the mean effects of treatments on the variable Average Daily Gemination. In turn, the results of the Tukey post hoc test, allow us to know that all treatments are in the same homogeneous group; that is, there was no significant difference between treatments. In the same way, the *p*-value of significance of the unifactorial ANOVA test reached a value greater than 0.05 (*p* = 0.843 > 0.05). There was no significant difference between the mean effects of treatments on the variable Czabator Index (CI). Thus, the results of Tukey’s post hoc test allow us to know that all treatments are in the same homogeneous group; there was no significant difference between treatments.

## 4. Discussion

Regarding the germination curve of *L. esculentum* (Figure 2 and Figure 3), it is evident that germination on average starts from day 6. This is due to the fact that the speed of germination is conditioned by the energy germination and by environmental factors, such as the humidity and temperature. It is worth mentioning that a germinated seed is considered to be that which shows visible root development; therefore, treatment 3 reflects a higher percentage of germination of *L. esculentum*, which in turn, shows the highest values of the mean daily germination (MDG) and Czabator index (CI)—both indicators of germinative vigor, which is synonymous with speed and uniformity of germination [36,37].

When statistically analyzed (*p* = 0.54 > 0.05), the non-existence of significant differences between the treatments was identified for the percentage of germination, average daily germination (MDG) and Czabator index (CI) (Figure 4). This is an indicator that there is no evidence of an effect of zinc oxide nanoparticles (ZnO NPs) on the germination percentage of *L. esculentum*. These results are based on the size of the nanoparticle. Investigations by Tarafdar et al., 2012 and Lira et al., 2018 [38,39] maintained that the use of nanoparticles between 5 and 20 nm had a greater ability to penetrate and move via plasmodesmata, compared to 30 nm nanoparticles, which were used during the experimental phase.

It is also important to note that the seed coat plays an important role in protecting the embryo from external factors that may cause damage, in turn, the coat may have selective permeability [40], which possibly explains the non-significant difference in germination based on the variation of the ZnO NP colloid concentration but not associated with phytotoxicity, since the same germination, radicle emergence and root elongation are considered widely used phytotoxicity tests due to advantages, such as their low cost [41,42]. Nevertheless, there is an influence of NPs on emergence. Other authors confirmed the promising effect of ZnO NPs on germination parameters in other types of seeds [43,44].

According to the emergence curve of *L. esculentum* (Figure 2, Figure 3 and Figure 4), this start occurred from day 12, when the elongation of the epicotyl and hypocotyl was found, making the cotyledons visible. Therefore, the true success that guarantees the growth and development of a seedling is determined by the emergence [8]. After the hard and impenetrable seed coat is broken, high enzymatic and metabolic activity is required for cell elongation [45]. When statistically analyzing the emergence (*p* = 0.021 < 0.05), the existence of significant differences was identified, with treatment T6 $\left(\bar{X}=73.333\right)$ having the highest average and demonstrating the existence of the effect of the ZnO NPs on the percentage of emergence of *L. esculentum*.

It is worth mentioning that zinc is an essential element for plants since it promotes the enzymatic and hormonal activity of the plant. In turn, zinc stimulates the synthesis of tryptophan, which stimulates the genesis of auxins, cytokinins and gibberellins, all of them hormones involved in the growth and development of plants [46,47]. Intriago’s research [48] affirmed that the use of zinc stimulates the development of new tissues, contributing to the growth and development of the seedling [49,50,51]. Amooaghaie, R. [52] evaluated the influence of different concentrations of Zn and ZnO (obtained by inorganic route) on tomatoes and wheat, where the results showed the effect on the germination process in seeds and even the improvement of growth parameters when colloids were applied at low concentrations.

On the contrary, the NPs decrease these characteristics when applied at high concentrations. This did not happen with this research, since the highest concentration (T6 = 99.076 ppm) was the one that has generated the best degree of emergence, due to the high degree of biocompatibility in using plant extracts, which coats the nanoparticles with plant metabolites and radicals (OH), thus, improving absorption and providing the capacity for a better emergence process.

During the evaluation of zinc absorption in stem plus foliage, a higher average was observed in treatment 2 (T2) (0.264 ppm), which, when statistically analyzed using the Welch’s ANOVA test and the post hoc test of Games–Howell (Figure 4), corroborates the existence of statistically significant differences between the treatments. However, when comparing these results with the reports of Sturikova, 2018 [53], it is evident that, in the experiment, there would be no direct relationship between the concentration evidenced in the root vs. stem plus foliage because the distribution of the nanoparticles must show an acropetal movement in the plant tissues. The results obtained are justified because the zinc oxide nanoparticles were retained in the seed coat and in the substrate. Even when the *L. esculentum* seedling did not finish emerging, they first came into contact with the radicle and then with the epicotyl during the elongation of the embryonic axes [8,54,55].

Research by Esper, 2020 [50] suggests that concentrations between 70 and 100 mg·L^−1^ of zinc oxide nanoparticles are optimal to significantly stimulate the emergence, root length and total production of fresh and dry biomass of the Zea mays crop “corn”. These results are due to the fact that zinc nanoparticles increase cell division and the activity of enzyme nitrate reductase, favoring root development [56,57,58]. In turn, it is worth mentioning that zinc oxide nanoparticles contribute to the productivity and tolerance to biotic and abiotic stress, since they have the ability to activate the antioxidant system in plants grown in areas with water limitations [59]. Antioxidant enzymes, such as catalase and peroxidase, are responsible for reducing biotic and abiotic stress [60,61].

**Figure 5:**
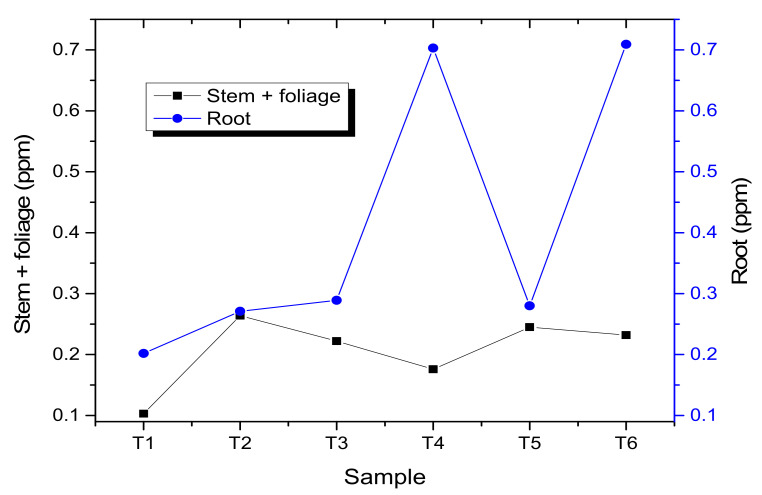
Quantification by atomic absorption for Zinc present in stem + foliage and root, as an influence of the application of ZnO NP in the emergence process.

As observed in the previous statistical results, a significant effect was shown in the emergence process for the T6 sample with 73.3% of seedling development, which implies that there was a positive effect of the ZnO NPs. However, it is important to highlight the interaction behavior between the nanoparticulate material and the seeds, in addition to the quantification of elemental zinc found later in the samples. In this sense, Figure 5 shows the results by atomic absorption evaluated in the stem+foliage and root sample groups achieved in the emergence process for zinc based on the concentration in ppm of ZnO NPs (T2, T3, T4, T5 and T6).

In this sense, it can be observed, in general terms, that all cases show higher zinc values compared with the control sample (T1), whose biological mechanism was described in the previous paragraphs. However, from the nanotechnological perspective, it is important to highlight that the ZnO NPs were obtained by the green synthesis methodology, using an organic extract of *C. sativum*, starting from the metal salt (zinc acetate dihydrate), which is a carrier of metal ions that in turn interacts with hydroxide ions (with negative charges) belonging to organic extract due to the presence of bioactive compounds, generating the reduction process, passing through growth phase and finally reaching the stabilization phase where Zn has a neutral charge.

This occurs due to a chelating effect in this phase, a covering with OH radicals (from the extract) is given to the neutral Zn atoms, forming a Zn^0^-phenolate complex, this latter allows the ZnO NP to possess OH-type free radicals which facilitate correct interaction between the seed and nanoparticle, showing no rejection processes due to possible toxicity symptoms, in this way positive effects of biosynthesized nanoparticle or obtained by green route methods are explained; however, the influence of concentration is considered for a better effect on the germinative characteristics discussed in the previous section.

It is important to consider in this description what is related to nanotoxicity, a subject that remains unknown, but studies point to the strong relationship with the chemical composition of the nanomaterial, in addition to its chemical structure, surface area and size [52]. Brunner, 2006 [62] attributed toxicity to two actions, chemical toxicity (linked to its composition) and the possible release of ions that are toxic, or related to the morphological characteristics of the nanoparticles (size, surface area, geometry) which cause stress. In this research, the ZnO NPs are scarcely soluble, but in spite of this, it has been absorbed by the radicle, and due to its small size (30 nm) it has been able to penetrate easily and motivate a positive influence on the emergence, with high biocompatibility, which reinforces the influence of practicing a green synthesis.

Despite the limitations, it is left open to continue with the line of research linked to evaluate with greater accuracy the mechanism of synthesis of nanoparticulate material from characterization by nuclear magnetic resonance (NMR), on the side of the application as nanofertilizer, evaluate if the same effect occurs in other types of seeds and thus have in ZnO NPs a material of universal application.

## 5. Conclusions

It has been determined that the extract of *C. sativum* possesses reducing properties of zinc metal salt and the formation of nanostructures, as determined by the FT-IR spectrum, which highlights the presence of bending at 429.5 cm^−1^ linked to the ZnO bond and bending at 3344 cm^−1^ corresponding to the (O-H) bond, which allows excellent biocompatibility for applications as nanofertilizers. In that sense, the results of the application of ZnO NPs indicate that concentrations close to 100 ppm of the NPs are ideal in the treatment of seeds of *L. esculentum* seeds, since it has shown an excellent promotion of enzymatic and metabolic activity to achieve cell elongation. In addition, the biosynthesized nanoparticles showed no phytotoxicity, since in all treatments there were germination and emergence processes. This is explained due to the presence of the hydroxyl radical, and the formation of the Zn^0^-phenolate complex by a chelating effect, which generates good compatibility with the seed providing it with improvements in the germination process.

Classical inorganic syntheses usually show phytotoxicity due to the presence of traces that are not biocompatible with organic agents, preventing germination. Therefore, this technology of green synthesis of nanoparticles is presented as a great alternative for this type of application, which deserves great attention due to the link with zero environmental impact compared to inorganic syntheses in addition to its great biocompatibility. Based on this interest and the diversity of applications, it is necessary to complement with more studies on the effects promoted by ZnO NPs, as well as studies linked to a framework of application in agriculture and even in food science.

## Sample Availability

Samples of the compounds are not available from the authors.
